# Dietary Antioxidants and Natural Compounds in Preventing Thrombosis and Cardiovascular Disease

**DOI:** 10.3390/ijms252111457

**Published:** 2024-10-25

**Authors:** Elvira Giurranna, Francesca Nencini, Alessandra Bettiol, Serena Borghi, Flavia Rita Argento, Giacomo Emmi, Elena Silvestri, Niccolò Taddei, Claudia Fiorillo, Matteo Becatti

**Affiliations:** 1 Department of Experimental and Clinical Biomedical Sciences “Mario Serio”, University of Firenze, 50134 Firenze, Italy; elvira.giurranna@unifi.it (E.G.); francesca.nencini@unifi.it (F.N.); alessandra.bettiol@unifi.it (A.B.); serena.borghi@unifi.it (S.B.); flaviarita.argento@unifi.it (F.R.A.); niccolo.taddei@unifi.it (N.T.); 2 Department of Medical, Surgery and Health Sciences, University of Trieste, 34100 Trieste, Italy; giacomo.emmi@units.it; 3 Department of Experimental and Clinical Medicine, University of Firenze, 50134 Firenze, Italy; elena.silvestri@unifi.it

**Keywords:** diet, antioxidants, natural compounds, oxidative stress, thrombosis, cardiovascular disease

## Abstract

Reactive oxygen species (ROS) contribute to endothelial dysfunction, platelet activation, and coagulation abnormalities, promoting thrombus formation. Given the growing interest in non-pharmacological approaches to modulate oxidative stress, we examine the potential of various dietary interventions and antioxidant supplementation in reducing oxidative damage and preventing thrombotic events. Key dietary patterns, such as the Mediterranean, Dietary Approaches to Stop Hypertension (DASH), and ketogenic diets, as well as antioxidant-rich supplements like curcumin, selenium, and polyphenols, demonstrate promising effects in improving oxidative stress markers, lipid profiles, and inflammatory responses. This review highlights recent advances in the field, drawing from in vitro, ex vivo, and clinical studies, and underscores the importance of integrating dietary strategies into preventive and therapeutic approaches for managing thrombosis and cardiovascular health. Further research is needed to better understand long-term effects and personalize these interventions for optimizing patient outcomes.

## 1. Introduction

Thrombosis is a main cause of death globally representing the most common underlying pathology in major cardiovascular (CV) events, including acute coronary syndrome, stroke, myocardial infarction, pulmonary embolism and venous thromboembolism. It occurs when a blood clot, or thrombus, forms within arterial or venous blood vessels due to an imbalance between pro- and anti-coagulant factors, leading to disrupted blood flow [1,2,3,4,5].

Arterial and venous thrombosis can be triggered by diverse factors such as trauma, non-traumatic insults, or various clinical disorders and is described by the three primary factors that contribute to the development of a thrombus which encompasses endothelial damage, impaired blood flow or stasis, and pro-thrombotic alterations originating from platelets and plasma components (Virchow’s triad) [6].

The development of thrombosis is influenced by a combination of genetic predispositions and acquired risk factors. Common genetic variants linked to thrombosis risk involve mutations in coagulation factors, coagulation inhibitors, the protein C system, the fibrinolytic pathway, and other non-hematological genes. Acquired risk factors include lifestyle factors, such as the use of certain medications, venous catheterization, surgeries, prolonged immobility, obesity, acquired disorders of hypercoagulation, cigarette smoking addiction, older age, and cancer. These factors disturb the hemostatic balance, increasing the likelihood of thrombus formation [7].

Among the causes of thrombosis, oxidative stress plays a pivotal role. Several studies have shown that both thrombus formation and its resolution may be regulated by reactive oxygen species (ROS) [2,8,9,10]. Indeed, elevated oxidative stress markers have been found in various thrombotic disorders and may serve as valuable prognostic indicators and therapeutic targets in patients with thrombotic complications [11]. Understanding the pathophysiology of thrombosis is crucial for the development of targeted preventive and therapeutic interventions for improving clinical outcomes in patients at risk of CV disorders (CVD) [12].

In addition to pharmacological interventions, growing evidence supports the role of specific nutritional diets and/or supplementations for reducing oxidative stress and, thus, the risk of CVD.

This review focuses on the strengths and weaknesses of studies regarding the potential efficacy of different type of diets or antioxidant supplementation for the prevention of thrombosis and CVD.

Our selection process involved a comprehensive literature search using databases such as PubMed, Scopus, and Web of Science. We focused on studies that provided new insights into the role of diet and dietary supplements. Priority was given to recent publications that offered new data or interpretations not covered in previous reviews. This narrative review summarizes the results of in vitro, ex vivo, and clinical studies highlighting significant advances and ongoing debates on the potential role of diet in the prevention of thrombotic events.

## 2. Oxidative Stress and Thrombosis

Oxidative stress is a condition characterized by an imbalance between excessive production and inadequate clearance of ROS, leading to disruption of the cellular redox balance [13].

**Figure 1:**
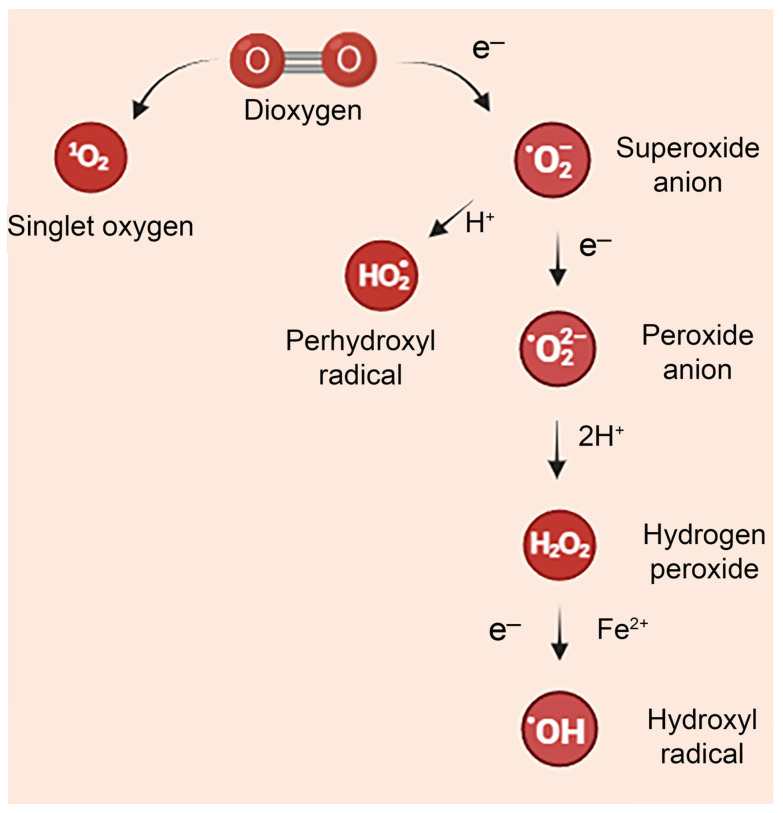
Chemical conversion of oxygen molecule to several ROS.

Molecules involved in regulating redox states include mainly ROS but also reactive nitrogen species (RNS), reactive sulfur species (RSS), reactive carbon species (RCS), and reactive selenium species (RSeS). These can consist of free radicals or non-radical species [14]. The primary ROS include the superoxide anion (O_2_^−•^), hydroxyl radical (OH^•^), peroxyl radical (ROO^•^), and alkoxyl radical (RO^•^). Other oxygen-derived molecules, although not being free radicals, are highly reactive and unstable, such as hydrogen peroxide (H_2_O_2_) and organic hydroperoxide (ROOH). RNS mainly include two species, nitric oxide (NO) and peroxynitrite anion (ONOO^−^) (Figure 1).

Free radical formation occurs through essential metabolic processes in the human body, such as mitochondria or enzymatic reactions involving hypoxanthine and xanthine oxidase, nicotinamide adenine dinucleotide phosphate (NADPH) oxidase, and the cytochrome P-450 system, as well as from external sources such as X-ray exposure, ozone, smoking, pollutants, and chemicals. To counteract the damaging effects of reactive species, the body has an antioxidant defense system, which includes enzymes and endogenous compounds like uric acid, glutathione, and melatonin. Additionally, exogenous antioxidants such as β-carotene, ascorbate and polyphenols contribute to this defense [15,16].

ROS are involved in physiological processes such as cell signaling, immune responses and aging. For a particular example, we can consider the NO molecule, which is a very important messenger in the vasodilation process [13,14]. However, ROS can also exert detrimental effects on the main biological macromolecules if exceeding antioxidant defense mechanisms. Excessive ROS production damages many molecules: DNA, resulting in mutations and cell death; proteins, whose aminoacidic sequence modifications significantly impacts their function; and lipids, which undergo a peroxidation process.

Also, endothelial cells, platelets and red blood cells (RBCs) are ROS targets, which impair their normal functions and lead to endothelial dysfunction and platelet activation [2,8,13], thus promoting thrombus formation [17,18].

### 2.1. Effects of ROS on the Endothelium

Endothelial cells are essential for maintaining the smooth flow of blood by creating a surface that discourages platelet activation and the clotting process [19]. Additionally, these cells have various properties that help dissolve clots and prevent their formation, ensuring uninterrupted blood circulation [20,21]. Moreover, endothelial cells produce nitric oxide (NO), which is a crucial bioactive substance. NO exerts a clear inhibitory influence on thrombosis by enhancing the synthesis of cyclic guanosine monophosphate and preventing platelet activation and aggregation [22]. Endothelial cells could react to both physical and chemical signals by releasing factors that control cell adhesion [23]. When these cells become dysfunctional, it heightens the likelihood of thrombosis [20].

Oxidative stress has been identified as a significant contributor to endothelial dysfunction [8,21,24,25,26,27,28,29,30,31]. ROS can cause protein modifications in endothelial cells, leading to structural and functional impairment [32]. This impairment can elevate the tendency of blood vessels to develop thrombi, in part because of compromised endothelium-dependent vasodilation [2]. This determines turbulent blood flow within the vessel, which is a critical factor contributing to thrombus formation [33].

Moreover, oxygen free radicals can interact with NO, forming peroxynitrite (ONOO^−^), which possesses both oxidizing and nitrifying properties [34]. This compound can exacerbate damage to cell membranes, proteins, and DNA while also diminishing the availability of NO, exacerbating the risk of thrombosis [2,35,36].

### 2.2. Effects of ROS on Platelets

Platelets serve as a critical cellular element in blood; they are primarily tasked with maintaining hemostasis and initiating thrombosis. Excess ROS can cause drastic changes in platelet metabolism and further affect platelet function. It will also lead to an increase in platelet procoagulant phenotype and cell apoptosis, which will increase the risk of thrombosis [37].

Upon vascular injury, platelets swiftly adhere to the damaged vessel wall, fostering further platelet aggregation and the formation of an initial thrombus. Following the aggregation of this primary thrombus, the phospholipids and tissue factor (TF) present on the platelet surface efficiently catalyze thrombin formation, facilitating fibrin production and enhancing thrombus stability. Additionally, platelets can release various chemical signaling molecules, contributing to inflammation and immune responses, including the induction of leukocyte migration and activation [38].

Platelets possess a robust antioxidant enzyme system, but an imbalance between the production of ROS and the efficacy of this antioxidant system can contribute to the development of thrombotic diseases [39]. Elevated intracellular ROS levels can result from this imbalance, consequently promoting increased platelet activation [40]. During activation, platelets themselves generate ROS, which in turn exacerbate platelet activation signaling pathways, leading to enhanced platelet aggregation, shape alteration, and the release of granules. Furthermore, high ROS levels can amplify the production of inflammatory mediators by platelets, such as platelet activating factor and thromboxane A2, further intensifying platelet activation and promoting thrombosis. Additionally, ROS can regulate the expression of platelet adhesion molecules, augmenting platelet adhesion [38,41].

Hence, ROS can indirectly heighten platelet reactivity by hindering endogenous mechanisms tasked with platelet inhibition. For instance, ROS can compromise the NO produced by endothelial cells, which typically exerts an anti-platelet aggregating effect. Moreover, ROS can impact calcium signaling within platelets, which is a crucial process in platelet activation [8,42].

### 2.3. Effects of ROS on RBCs

RBCs play a role in blood clotting: an inverse correlation exists between hematocrit levels and bleeding time; conversely, abnormally high hematocrit, as seen in conditions like polycythemia vera, is associated with an increased blood viscosity and a higher thrombosis risk [43].

Also, changes in the RBC structure and function can also contribute to a pro-thrombotic state [44]. Diseases like sickle cell disease and thalassemia, as well as chronic conditions like diabetes and hypertension, can make RBCs less deformable, impacting their ability to pass through small blood vessels and increasing platelet margination [9,45,46]. Furthermore, under certain conditions, RBCs may release microparticles that enhance thrombin generation and systemic inflammation. Structural changes in erythrocytes, such as the exposure of phosphatidylserine on their surface, can also promote thrombosis [47].

RBCs can directly influence clot structure, with evidence suggesting their integration into thrombi, particularly in conditions where erythrocyte structure is altered [48]. This integration affects fibrin network formation and clot properties [9,17].

RBCs produce high quantities of ROS, mostly by NADPH oxidase activation and hemoglobin autoxidation, and can uptake and accumulate extracellular ROS [49]. On their turn, ROS can accelerate hemolysis and induce a hypercoagulable state [50]. Indeed, ROS can alter RBCs membrane structure, function, and promote RBCs lysis, thus inducting RBCs binding to endothelial cells and the activation of platelet and of coagulation factors [51]. Also, ROS induce the exposure of phosphatidylserine and the release of microvesicles as well as the adherence of RBCs to the vessel wall [52].

Interestingly, RBCs may also have anti-thrombotic properties. For instance, deoxygenated hemoglobin can stimulate the release of NO, which inhibits platelet reactivity [53].

### 2.4. Effects of ROS on Coagulation and Inflammatory Factors

In the presence of oxidative stress, there is an elevated expression of key adhesion molecules such as Intercellular Adhesion Molecule 1 (ICAM-1) and Vascular Cell Adhesion Molecule 1 (VCAM-1). These molecules, part of the superfamily of cell adhesion molecules, play crucial roles in mediating interactions between leukocytes and endothelial cells. The increased expression of ICAM-1 and VCAM-1 can augment platelet adhesion to the endothelium, potentially culminating in thrombosis [54]. Additionally, ROS upregulate TF expression in endothelial cells. Although endothelial cells also produce TF pathway inhibitor (TFPI), the primary physiological regulator of TF activity, oxidative stress can inhibit TFPI, inducing a procoagulant effect [2,55].

Studies have indicated that inflammatory stimuli can also diminish the levels of thrombomodulin (TM) in endothelial cells. TM is a protein located on the membranes of endothelial cells. It serves as a crucial mechanism by which the endothelium manages hemostasis. TM interacts with thrombin, either binding to and sequestering it or enhancing its affinity for protein C, which is an anti-coagulant factor [56,57].

Concomitantly, oxidative stress can trigger alterations in the expression of inflammation-related genes within endothelial cells. Among these genes, nuclear factor-kappa B (NF-κB) stands out as a critical regulator of inflammatory signaling pathways. The activation of NF-κB in endothelial cells promotes the expression of various inflammatory factors, including tumor necrosis factor-alpha (TNF-α), interleukin-6 (IL-6), and Monocyte Chemoattractant Protein-1 (MCP-1) [58]. The elevation of these inflammatory factors facilitates the migration of leukocytes to the vascular endothelium and enhances the adhesion of endothelial cells to platelets, heightening the risk of thrombus formation [2,11,13]. The stimulation of endothelial cells with pro-inflammatory cytokines such as TNF-α and IL-1 leads to the upregulation of TF and von Willebrand factor (vWF) production while concurrently reducing the expression of TM, NO, and prostacyclin (PGI2) [59]. Notably, a reduction in PGI2 levels indicates a compromised anti-thrombotic defense function.

Inflammatory mediators, such as cytokines and chemokines, can activate endothelial cells and leukocytes, promoting a pro-thrombotic state [41]. Among white blood cells, neutrophils play an important role in the process of thrombosis and have garnered increasing attention in thrombosis research [60]. Neutrophils contribute to the pathogenesis of thrombosis by the extrusion of web like structures known as neutrophil extracellular traps (NETs), which consist of decondensed chromatin and antimicrobial proteins [61]. NETs recently emerged as a newly recognized contributor to venous and arterial thrombosis. These strands of DNA, extruded by activated or dying neutrophils, decorated with various protein mediators, become solid-state reactors that can localize at the critical interface of blood with the intimal surface of diseased arteries alongside propagating and amplifying the regional injury. NETs thus furnish a previously unsuspected link between inflammation, innate immunity, thrombosis, oxidative stress, and cardiovascular diseases [62]. In response to disease-relevant stimuli, neutrophils undergo a specialized series of reactions that culminate in NET formation [63]. Moreover, similarly to ROS, also NETs have been shown to promote a procoagulant state in animal models and in humans, and they contribute to some arterial diseases such as stroke and myocardial infarctions [64,65].

These biochemical processes are complex, and only a part of them have been described. Indeed, there are many molecules involved in thrombus formation, and pathways that connect these processes have not yet been fully elucidated.

### 2.5. Effects of ROS on Fibrin(ogen) Structure and Function

Fibrinogen is one of the main molecular players in hemostasis. It is a precursor of fibrin, which is a major protein component of intravascular thrombi in all locations; concomitantly, the conversion of fibrinogen to fibrin exposes binding sites for fibrinolytic proteins to limit clot formation and avoid the unwanted extension of fibrin fibers [66,67]. A wide variety of factors can modulate fibrin properties, such as multiple mRNA transcripts (generated by initiation of transcription by alternative promoters, differential termination of transcription, alternative mRNA splicing, or genetic recombination), environmental factors, post-translational modifications (PTMs) of fibrinogen and pathological conditions [68,69,70,71,72,73,74,75]. These factors can impact on fibrin susceptibility to lysis by plasmin, making fibrin networks more resistant to lysis and thus increasing the risk of thrombosis, or making the fibrin clot more susceptible to lysis and therefore weak and unstable, increasing the risk of bleeding [66,76].

Among these factors, PTMs exponentially amplify the complexity and heterogeneity of fibrinogen and clot structure, modifying the fibrinogen molecule in many ways, such as phosphorylation at specific seryl and threonyl sites, prolyl hydroxylation, tyrosyl sulfation, asparaginyl or glutaminyl deamidation, N-terminal pyroglutamate formation from glutaminyl precursors, oxidation of methionine, histidine and tryptophan residues, tyrosine nitration, modifications of cysteine residues, and the formation of dityrosine and carbonyl groups [71,77,78].

Several in vitro and in vivo studies have paid increasing attention to the post-translational modifications of fibrinogen and its effect on clot formation [69,79,80,81,82,83,84,85,86,87,88,89,90,91,92].

Oxidation and nitration, closely linked to oxidative stress, influence fibrin fiber formation by generating more compact and resilient fibrin networks, which can exacerbate pro-thrombotic conditions such as cardiovascular diseases and chronic inflammatory disorders. Glycosylation and glycation modify fibrinogen’s structural properties, often leading to changes in clot density and resistance to lysis. Conversely, modifications like acetylation, often induced through aspirin therapy, result in more permeable clots with thicker fibers, enhancing fibrinolytic susceptibility.

All these effects have important consequences for the occurrence and progression of thrombotic diseases [18].

## 3. Diet or Supplementation and Oxidative Stress

### 3.1. Pro-Oxidant Diets

Given the well-established central role of oxidative stress in cardiovascular diseases (CVDs), targeting oxidative stress pathways has emerged as a promising strategy for preventing and managing these conditions. Recent evidence highlights the influence of lifestyle factors on the complex interplay between oxidative stress, inflammation, thrombosis, and overall CVD risk. In particular, lifestyle modifications such as regular exercise and a healthy diet have been shown to reduce oxidative stress and lower thrombotic risk [93,94].

Several studies have observed that the long-term consumption of diets high in fatty acids can shift oxidative stress toward a pro-oxidant state in both animal models and human subjects. For instance, research has indicated that fatty acid-rich diets lead to changes in the expression of novel proteins, cellular ligands, and inflammation markers [95,96,97].

In patients with metabolic syndrome (MetS)—a multi-component disorder associated with a high risk of CVD—67 proteins were found to be differentially expressed after the long-term consumption of four different diets. A diet high in saturated fatty acids specifically increased the expression of proteins linked to oxidative stress, ubiquitinated protein degradation, and DNA repair. In contrast, the other three diets (rich in monounsaturated fatty acids; low-fat, high-complex carbohydrate with a placebo supplement; and low-fat, high-complex carbohydrate with long-chain n-3 polyunsaturated fatty acids (PUFAs)) were associated with a reduction in pro-inflammatory proteins related to oxidative stress and DNA repair. These findings suggest that a diet high in saturated fatty acids may increase CVD risk factors associated with MetS, such as inflammation and oxidative stress, and contribute to DNA damage as a consequence of elevated oxidative stress [95].

Further supporting this, another study on mice subjected to a high-fat diet for 16 weeks demonstrated significant increases in oxidative stress markers (NADPH oxidase expression, dihydroethidium fluorescence) and inflammatory parameters (inducible nitric oxide synthase, interleukin-6 expression) in wild-type mice with implications for similar effects in humans [96].

Additionally, Geys et al. confirmed that diet, rather than genotype, influenced the expression of inflammation and oxidative stress markers in their study on mice [97]. A separate study using a rat model revealed that a high-fat, high-cholesterol (HFHC) diet resulted in systemic and cardiac lipid dysregulation, accompanied by oxidative and pro-inflammatory stress, which contributed to pathological changes in heart tissue, suggesting that maintaining lipid regulation is essential for preventing heart damage [98].

Animal-based diets have also been shown to elevate CVD risk by increasing oxidative stress and cardiovascular inflammation through factors such as enhanced toll-like receptor (TLR) signaling, cardiovascular lipotoxicity, and elevated serum trimethylamine-N-oxide levels, independent of one another. This underscores the importance of public health policies advocating for a primarily plant-based diet and minimizing animal-based food consumption [99]. Moreover, low-carbohydrate, animal-based diets inherently lead to higher saturated fatty acid intake, resulting in predictable increases in serum LDL cholesterol levels [100,101].

Fish consumption has been linked to reduced mortality and lower CVD incidence [102,103]. Interestingly, population studies in the United States revealed a U-shaped mortality curve with an optimal fish intake of around 20 g/day, while higher consumption was associated with increased mortality [104]. In contrast, Japanese populations showed a more linear relationship between fish intake and reduced CVD mortality, suggesting potential differences in preparation methods. Fish offers omega-3 fatty acids and lower saturated fat content, providing protective effects; however, it also contains carnitine and choline at higher concentrations than plant-based foods, indicating that fish intake should be monitored [105].

### 3.2. Antioxidant Diet

The Mediterranean diet (MD) is widely recognized as one of the most studied dietary patterns with numerous randomized controlled trials, meta-analyses, and systematic reviews consistently supporting its positive effects on cardiovascular health [106,107,108,109,110]. This diet is characterized by a high intake of fruits, vegetables, whole grains, legumes, nuts, seeds, and fish alongside a moderate consumption of saturated fats and limited intake of red meats and dairy products. Moderate wine consumption, particularly red wine, is often included in the MD and is thought to contribute to its health benefits due to its rich polyphenol and antioxidant content [111].

The MD has been linked to various health benefits, such as enhanced heart health, reduced risks of chronic conditions like diabetes and obesity, and improved longevity. Importantly, it has shown a substantial impact on oxidative stress, which is a key contributor to CV diseases. For instance, a 2007 study found that individuals at high CV risk who shifted toward an MD demonstrated notable reductions in cellular lipid levels and LDL oxidation [112]. Similarly, research by Yubero-Serrano et al. indicated that the MD more effectively modulates endothelial function and intracellular ROS production than a low-fat diet in coronary artery disease patients, even among those with severe endothelial dysfunction [113]. Additionally, a year-long MD intervention increased plasma total antioxidant capacity (TAC) in subjects at high CVD risk, underscoring its antioxidant potential [114].

The benefits of the MD extend beyond traditional cardiovascular risk factors, as evidenced by studies involving other health conditions. In Behçet’s syndrome (BS) patients, dietary interventions such as a lacto-ovo-vegetarian diet or supplementation with oral butyrate significantly reduced ROS production in leukocytes and plasma lipid peroxidation while enhancing plasma TAC [115,116]. Similarly, in patients with endometriosis, a lifestyle improvement incorporating the MD contributed to improved metabolic and oxidative profiles and enhanced quality of life [117]. Moreover, in the context of atrial fibrillation, the MD has been shown to reduce cardiovascular events and oxidative stress, partly by favorably modulating the antioxidant activity of glutathione peroxidase 3 (GPx3), leading to a lower rate of vascular events [118,119]. Interestingly, comparisons of different diets, such as in the study by Sofi et al., suggest that both a low-calorie lacto-ovo-vegetarian diet and the MD can significantly improve oxidative stress parameters compared to a control diet even if no significant differences were observed between the two [120]. In parallel, other dietary patterns, such as the ketogenic diet (KD), have demonstrated potential benefits in modulating oxidative stress and inflammation. The KD, characterized by high fat and low carbohydrate intake, increases blood β-hydroxybutyrate (β-HB) levels, which possess ROS-scavenging properties [121,122,123,124]. It appears to enhance antioxidant defenses through various mechanisms, such as an increased production of superoxide dismutase (SOD-I and II) and NADPH quinone dehydrogenase 1 (NQO1), alongside enhancing glutathione (GSH) synthesis [125,126,127]. This combination of effects may help improve mitochondrial function, activate protective antioxidant pathways, and lower oxidative stress. Evidence also suggests that a vegetarian diet, rich in antioxidants, contributes to cardiovascular health, potentially due to its role in reducing oxidative stress [128,129,130,131,132,133]. Comparative studies have shown that omnivorous diets, associated with higher ROS and nitric oxide production, trigger a compensatory increase in heme-oxygenase-1 (HO-1), a protective response to oxidative stress, while such activation is not observed in vegetarians [134]. However, Peluso et al. concluded that there is no conclusive evidence on the role of vegetables in modulating antioxidant status markers [135]. Antioxidant-rich dietary interventions have demonstrated beneficial effects on oxidative stress and metabolic health. For instance, a 4-week intervention in elderly Koreans with Metabolic Syndrome (MetS) led to improvements not only in oxidative stress but also in various aspects of MetS, such as central obesity, dyslipidemia, hypertension, and arterial stiffness [136]. Similarly, a study by Rossi et al., utilizing a food frequency questionnaire, emphasized the importance of diet in boosting non-enzymatic antioxidant capacity (NEAC) for the prevention of acute myocardial infarction (AMI), advocating for a diet rich in fruits, vegetables, moderate wine intake, and whole grains [137].

Other antioxidant-rich diets, such as the DASH diet, have similarly demonstrated the ability to manage oxidative stress [138,139,140]. A meta-analysis revealed that the DASH diet significantly improved antioxidant markers, including GSH, TAC, and nitric oxide, while reducing oxidative stress indicators like MDA and f2-isoprostanes [141]. Although these findings are promising, further studies and randomized clinical trials are necessary to confirm the DASH diet’s effects on oxidative stress parameters.

In summary, these dietary patterns, particularly the Mediterranean, ketogenic, vegetarian, and DASH diets, share common elements in their ability to modulate oxidative stress and inflammation, highlighting the importance of dietary interventions in managing cardiovascular risk and enhancing overall health.

### 3.3. Role of Dietary Supplements in Modulating Oxidative Stress

In recent years, there has been a notable rise in the use of dietary supplements to fulfill nutritional needs, reflecting the growing recognition that certain nutrients are challenging to obtain solely through diet [142,143]. This has underscored the importance of supplementing with essential vitamins and minerals, which are available in various forms such as tablets, capsules, powders, gels, and liquids [144,145]. Dietary supplements have proven effective in restoring intracellular antioxidants, aiding in the neutralization of oxidative damage, and supporting cardiometabolic health.

Research has increasingly focused on whether supplementation with naturally derived antioxidants, such as sweet potato leaf powder, gualoupi, flaxseed oil, onion extract, and compounds like selenium, amino acids, carotenoids, and flavonoids, can influence oxidative stress [146,147,148,149,150,151]. These studies consistently demonstrated that such supplementation on top of habitual diet improves oxidative stress markers by reducing ROS, oxidative stress-related proteins, and inflammatory proteins while enhancing the total antioxidant capacity (TAC). Furthermore, evidence suggests that a diet rich in plant-derived compounds, especially (poly)phenols, plays a significant role in promoting cardiovascular health [152].

#### 3.3.1. Supplementation with Bioactive Compounds

Selenium, though present in trace amounts in the human body, plays a vital role in numerous cellular functions, particularly in supporting intracellular antioxidant enzymes like GPx and thioredoxin reductase [153]. Its dietary intake is crucial, as selenium has demonstrated a positive impact on cardiovascular health. However, at high doses, it can be toxic; in fact, a dose in the range of 200/300 µg per day was used in the studies reviewed. For instance, in individuals with chronic venous disease, selenium supplementation significantly reduced oxidative stress, as measured by the free oxygen radicals defense (FORD) and free oxygen radicals (FORT) tests [154]. Another study involving elderly individuals living in the community showed that supplementation with selenium and coenzyme Q10, known for its protective effects against lipid peroxidation, led to a reduction in two biomarkers: copeptin, a surrogate for vasopressin, and adrenomedullin, which is an indicator of oxidative stress in tissues [155].

Animal studies further support selenium’s benefits, where its supplementation in the diet of maneb-treated mice showed a remarkable protective effect against cardiotoxicity. This was evident as it counteracted the oxidative damage, lipid and protein oxidation, and disruption of antioxidant status induced by the fungicide, preserving heart histoarchitecture [156]. In broiler chickens, dietary supplementation with 0.30 mg/kg of nano-selenium was effective in preventing ventricular hypertrophy and reducing lipid peroxidation in the liver [157].

Melatonin, another important dietary component available as a supplement, has shown significant cardiometabolic benefits due to its strong antioxidant properties [158,159]. Administering 10 mg of melatonin daily for three months in patients with type 2 diabetes (T2D) and coronary heart disease resulted in improved metabolic and lipid profiles, lowered blood pressure, and reduced inflammation and oxidative stress markers, such as malondialdehyde (MDA) and high-sensitivity C-reactive protein (hs-CRP), along with increased plasma GSH levels [160,161]. Furthermore, in patients with systemic lupus erythematosus (SLE), melatonin supplementation reduced oxidative stress, although it did not impact disease activity [162].

In addition to selenium and melatonin, other compounds like luteolin, chrysin, zinc, and methionine have also demonstrated potent protective effects against oxidative and inflammatory damage, further highlighting their potential role in mitigating oxidative stress-related conditions [163,164,165,166,167,168,169,170,171,172,173,174,175,176,177].

#### 3.3.2. Vitamin Supplementation

There is growing interest in the potential cardiovascular benefits of vitamin supplementation, as research increasingly links various vitamins to the modulation of cardiovascular risk factors. Vitamin E, in particular, has demonstrated significant antioxidant properties, with studies showing that supplementation, either alone or combined with vitamin C, can effectively reduce oxidative stress. For instance, in patients at high cardiovascular risk, a negative correlation was observed between malondialdehyde (MDA) levels and TAC [178]. Moreover, the combined supplementation of vitamins E and C has been shown to improve oxidative stress markers in women with endometriosis, which is a condition often associated with chronic inflammation [179].

The impact of vitamin E on cardiovascular health is further supported by a 2000 study published in The Lancet, which found that hemodialysis patients with cardiovascular disease exhibited higher oxidative stress compared to those without the disease. High-dose vitamin E supplementation (800 IU/day) led to improved cardiovascular outcomes, including a reduction in composite cardiovascular endpoints and myocardial infarction among these patients, suggesting a role for oxidative stress in the cardiovascular complications frequently observed in this population [180].

B vitamins have also shown promise in cardiovascular health, although fewer studies have specifically examined their impact on oxidative stress [181,182,183]. For example, Hagar et al. demonstrated that folic acid and vitamin B12 supplementation reduced myocardial cell damage, homocysteine levels, and oxidative stress in hyperhomocysteinemic rats [184]. Similarly, recent research found that pyridoxamine, a vitamin B6 analog, effectively alleviated interstitial fibrosis and oxidative stress in the hearts of prediabetic rats on a Western diet [185].

Overall, these findings underscore the therapeutic potential of antioxidant vitamins in preventing and managing cardiovascular diseases and other conditions related to oxidative stress. While further research is needed to establish definitive clinical guidelines, current evidence suggests that vitamin supplementation could play a beneficial role in reducing cardiovascular risk and alleviating symptoms of chronic inflammatory conditions, such as endometriosis [178,179,180,185,186].

#### 3.3.3. Supplementation with Natural Extracts and Compounds

The literature review highlights the therapeutic potential of a variety of natural extracts, such as thymol, spirulina, artichoke extract, delphinidin, maoberry, and mango peels, in the treatment and prevention of chronic diseases. These compounds exhibit significant effects in reducing oxidative stress, inflammation, and cellular damage, presenting promising options for managing conditions such as atherosclerosis, hypertension, non-alcoholic fatty liver disease (NAFLD), and dyslipidemia [187,188,189,190,191,192,193,194,195,196,197,198,199,200,201,202,203,204,205,206]. While there are limited studies on many of these extracts, curcumin stands out due to its well-documented antioxidant, anti-inflammatory, and free radical scavenging properties [207]. Some studies have explored curcumin, often in combination with piperine, as an antioxidant supplement to mitigate oxidative stress. For instance, Boshagh et al. (2023) found that curcumin–piperine supplementation significantly improved clinical parameters and increased TAC in stroke rehabilitation patients after 12 weeks [208]. Similarly, Helli et al. demonstrated that both curcumin and nano-curcumin supplementation led to notable improvements in lipid profiles, oxidative stress, and inflammatory markers in cardiac patients [207]. These findings are consistent with other studies showing that curcumin enhances metabolic health and reduces CVD markers [209,210,211]. In addition, natural oils such as wild olive, borage, coconut, sesame, and flaxseed have shown benefits in reducing oxidative stress, improving lipid profiles, and modulating systems like the renin–angiotensin system [212,213,214,215,216,217]. Particularly, olive oil, rich in monounsaturated fats and antioxidant phenolic compounds such as hydroxytyrosol and oleuropein, is renowned for promoting cardiovascular health, a key feature of the Mediterranean diet, while protecting DNA from oxidative damage [213,218,219,220,221].

## 4. Conclusions

**Figure 2:**
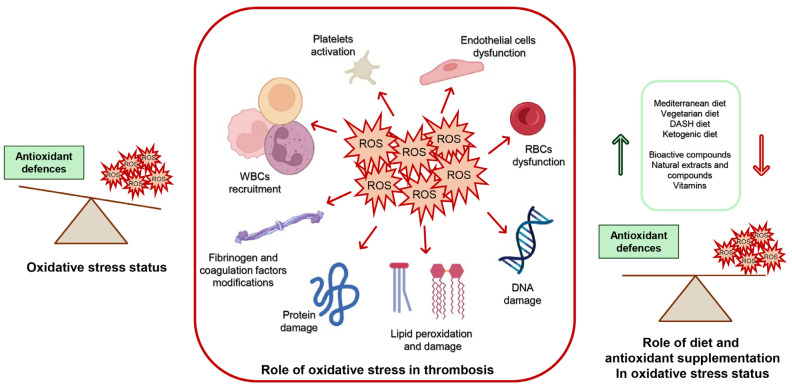
Impact of ROS production on molecular and cellular targets (illustrated with BioRender.com), disrupting their structure and function and promoting thrombus formation. The role of diet and antioxidant supplementation in modulating oxidative stress is highlighted. RBCs: red blood cells; WBCs: white blood cells.

In conclusion, the relationship between oxidative stress and thrombosis is well established, with ROS playing a pivotal role in endothelial dysfunction, platelet activation, and coagulation dysregulation, all of which contribute to thrombus formation (Figure 2). Targeting oxidative stress through dietary interventions and supplementation has emerged as a promising strategy for reducing cardiovascular risk and improving health outcomes. Diets rich in antioxidants, such as the Mediterranean and DASH diets, have shown significant benefits in lowering oxidative stress markers and enhancing cardiovascular health. Additionally, bioactive compounds like curcumin, selenium, and natural oils demonstrate potential in modulating oxidative pathways, improving lipid profiles, and preventing inflammation. The results of in vitro, ex vivo, and clinical studies, are summarized in Table 1.

While current research underscores the therapeutic value of these interventions, further studies are needed to better understand their long-term effects and to develop more personalized approaches for managing oxidative stress and preventing cardiovascular disease. Ultimately, integrating these dietary and supplementation strategies into clinical practice could provide a valuable complement to pharmacological therapies in managing thrombosis and cardiovascular conditions.

## Figures and Tables

**Table 1:** Impact of diets/supplementation on oxidative stress in studies analyzed.

Author	Model	Type of Diet/Supplementation	Oxidative Stress
***Diets with pro-oxidant effect***			
Rangel-Zúñiga et al. (2015) [95]	Metabolic syndrome patients	High-saturated fatty acid (HSFA)	↑
		High-monounsaturated fatty acid (HMUFA)	↓
		Low-fat, high-complex carbohydrate diets supplemented with placebo (LFHCCs)	↓
		Low-fat, high-complex carbohydrate diets supplemented with long chain (LC) n-3 polyunsaturated fatty acids (PUFA) (LFHCC n-3)	↓
Steven et al. (2018) [96]	Male wild-type and CD40L−/− mice	High-fat diet	↑
Han et al. (2018) [98]	Male Sprague–Dawley rats	High-fat high-cholesterol diet	↑
Cho et al. (2017) [105]	Healthy young men	Meals containing trimethylamine-N-oxide (TMAO) (fish)	↑
***Diets with anti-oxidant effect***			
Fitó et al. (2007) [112]	Subjects at high cardiovascular risk	Traditional Mediterranean diet	↓
Yubero-Serrano et al. (2020) [113]	Patients with coronary heart disease	Mediterranean diet	↓
Zamora-Ros et al. (2013) [114]	Patients with high cardiovascular risk	Mediterranean diet	↓
Emmi et al. (2021) [115]	Behçet’s syndrome (BS) patients	Butyrate-enriched diets	↓
Cirillo et al. (2023) [117]	Patients with endometriosis	Mediterranean diet	↓
Pastori et al. (2015) [118]	Atrial fibrillation patients	Mediterranean diet	↓
Pastori et al. (2016) [119]	Prospective cohort study	Mediterranean diet	↓
Sofi et al. (2018) [120]	Overweight omnivorous subjects with a low-to-moderate cardiovascular risk profile	Low-calorie lacto-ovo vegetarian diet	↓
		Low-calorie Mediterranean diet	↓
Xu et al. (2023) [124]	Adult Sprague–Dawley rats	Ketogenic diet	↓
Lu et al. (2018) [123]	Rats with spinal cord injury	Ketogenic diet	↓
Greco et al. (2016) [127]	Male rats	Ketogenic diet	↓
Cinegaglia et al. (2020) [134]	Healthy men subjects	Vegetarian dietsVS	↓
		Omnivorous diets	↑
Peluso et al. (2018) [135]	Subjects at risk of CV diseases	High intakes of vegetables	=
Chung et al. (2022) [136]	Elderly Koreans with metabolic syndrome	Antioxidant-rich dietary intervention	↓
Rossi et al. (2014) [137]	Patients below 75 years with a first episode of acute myocardial infarction	Food frequency questionnaire using Italian food composition tables	↓
Azadi-Yazdi et al. (2017) [138]	Women with polycystic ovary syndrome	DASH diet	↓
Foroozanfard et al. (2017) [139]	Women with polycystic ovary syndrome	DASH diet	↓
Asemi et al. (2014) [140]	Overweight and obese women with polycystic ovary syndrome	DASH diet	↓
***Antioxidant Supplementation***			
Groussard et al. (2021) [146]	Wistar rats	Linseed oil supplementation	↓
Ko et al. (2018) [147]	Sprague–Dawley rats	Methanol fractions (MFO) and flavonols extracted (quercetin and quercetin glucosides) from onion	↓
Thushara et al. (2014) [148]	Healthy donors	Crocin supplementation	↓
Dehghani et al. (2021) [149]	Post-MI patients	Quercetin supplementation	↓
Xia et al. (2024) [150]	Coronary heart diseases rat model	Trichosanthis pericarpium (TP; Gualoupi, pericarps of Trichosanthes kirilowii Maxim) supplementation	↓
Chang et al. (2021) [151]	Syrian hamsters	Sweet potato leaf powder	↓
**Selenium supplementation**			
Danciu et al. (2023) [154]	Individuals with chronic venous disease	Selenium supplementation	↓
Alehagen et al. (2022) [155]	Elderly community-living persons	Supplementation with selenium and coenzyme Q10	↓
Sefi et al. (2022) [156]	Mice with induced cardiotoxicity	Selenium supplementation	↓
Zamani Moghaddam et al. (2017) [157]	Broiler chickens	Selenium supplementation	↓
**Melatonin supplementation**			
Bazyar et al. (2021) [160]	Patients with type 2 diabetes mellitus	Melatonin supplementation	↓
Raygan et al. (2019) [161]	Diabetic patients with coronary heart disease	Melatonin supplementation	↓
Nabatian-Asl et al. (2021) [162]	Systemic lupus erythematosus (SLE) patients	Melatonin supplementation	↓
***Antioxidant Supplementation***			
Bingül et al. (2024) [163]	Guinea pigs with diet-induced non-alcoholic fatty liver disease	S-adenosylmethionine (SAM)	↓
Jayachandran et al. (2015) [164]	Hamsters fed an atherogenic diet	Geraniol supplementation	↓
Nabhani et al. (2022) [165]	Women with gestational diabetes mellitus	Synbiotic supplementation	↓
Suchal et al. (2016) [166]	Experimental model of isoproterenol-induced cardiac toxicity in rats	Kaempferol (KMP), dietary flavonoid, supplementation	↓
Dong et al. (2023) [167]	Sprague–Dawley rats with hyperlipidemia-induced cardiac damage	Luteolin supplementation	↓
Tayebi Khosroshahi et al. (2018) [168]	Hemodialysis patients	High amylose-resistant starch (HAM-RS2) supplementation	↓
Yuvaraj et al. (2022) [169]	Male Wistar rats	Chrysin supplementation	↓
Barman et al. (2017) [170]	Diabetic rats model	Zinc supplementation	↓
Alam et al. (2018) [171]	Aged Long Evans male rats	Astaxanthin supplementation	↓
Corsi et al. (2018) [172]	Pilot prospective observational study	Polyphenol-based multicomponent nutraceutical supplementation	↓
Lu et al. (2023) [173]	Apolipoprotein E-knockout mice	Oral ovatodiolide and antcin K (OAK) supplements	↓
Wang et al. (2020) [174]	Adult male SD rats	Chlorogenic acid supplementation	↓
Belcaro et al. (2015) [175]	Subjects without any conventional risk factors who had a stenosing atherosclerotic plaque	Nutritional supplements Pycnogenol^®^ and total triterpenic fraction of Centella asiatica (TTFCA)	↓
Belcaro et al. (2017) [176]	Low-risk, asymptomatic subjects with carotid or femoral stenosing plaques	Nutritional supplements Pycnogenol^®^ and Centella asiatica (CA)	↓
Belcaro et al. (2020) [177]	Asymptomatic patients with atherosclerotic plaques (Class IV and V) and arterial wall atherosclerotic lesions and intima-media thickening (IMT)	Supplementation with Pycnogenol^®^ + Centellicum^®^	↓
**Vitamin supplementation**			
Karajibani et al. (2010) [178]	CVD patients	Supplementation of vitamins E and C	↓
Amini et al. (2021) [179]	Women with endometriosis	Supplementation with antioxidant vitamins (Combined Vit C and Vit E)	↓
Boaz et al. (2000) [180]	Hemodialysis patients with pre-existing cardiovascular disease	High-dose vitamin E supplementation	↓
D’Haese et al. (2024) [185]	Rats who develop T2DM	Pyridoxamine, vitamin B6 analog, supplementation	↓
Hamdan et al. (2022) [186]	High-sucrose/fat (HSF) diet Wistar male rats	l-ascorbic acid supplementationVit C	↓
**Natural extracts and compounds**			
Yu et al. (2016) [187]	Hyperlipidemic rabbits	Thymol, major polyphenolic compound in thyme, supplementation	↓
Sun et al. (2022) [188]	Rabbits with atherosclerosis	Delphinidin-3-O-glucoside, active compound of Hibiscus sabdariffa calyces, supplementation	↓
Lu et al. (2021) [189]	Type 2 diabetic mice model	Hinokinin supplementation	↓
Ahmed-Farid et al. (2023) [190]	Hypertensive rats	Hordeum vulgare ethanolic extract	↓
Clemente et al. (2021) [191]	Overweight people with physical disability	Watercress extract supplementation	=
Martínez-Sámano et al. (2018) [192]	Patients with systemic arterial hypertension	Spirulina (Arthrospira) maxima supplementation	↓
Wang et al. (2019) [193]	High-salt-induced hypertensive mice	Xin-Ji-Er-Kang (XJEK), a Chinese herbal formula	↓
Deng et al. (2022) [194]	Rats with high-fat diet-induced non-alcoholic fatty liver disease	Water extract from artichoke	↓
Atkin et al. (2016) [195]	Adults with type 2 diabetes at high cardiovascular risk	Aged garlic extract (AGE) supplementation	=
Satheesh Babu et al. (2024) [196]	Mice	Blueberries supplementation	↓
		Strawberries supplementation	=
Kosuru et al. (2018) [197]	Sprague–Dawley rats	Pterostilbene, the primary antioxidant in blueberries	↓
Van der Werf et al. (2018) [198]	Type 2 diabetic rats	Cherry consumption	↓
Wang et al. (2019) [199]	Type 2 diabetic rats	Sanbai melon seed oil exerts	↓
Arshad et al. (2021) [200]	Overweight females subjects	Mango peels powder supplementation	↓
Udomkasemsab et al. (2018) [201]	Rats fed a high-fat diet	Maoberry (Antidesma bunius), antioxidant-rich tropical fruit, supplementation	↓
Mokhtari et al. (2023) [202]	Mice	Loquat fruit peel extract supplementation	↓
Halima et al. (2018) [203]	High-fat-fed male Wistar rats	Apple cider vinegar	↓
Sierra et al. (2022) [204]	Adult men	Golden berry (Physalis peruviana), tropical fruit rich in antioxidants	↓
Feriani et al. (2021) [205]	Atherogenic diet-induced obese rats	Schinus terebinthifolius fruits extract supplementation	↓
Giannenas et al. (2022) [206]	Young layers	Dietary supplementation with a phytonutrient solution (PHYTO) plant extract combination of *Scutellaria baicalensis* and *Curcuma longa*	↓
Helli et al. (2021) [207]	Patients undergoing coronary elective angioplasty	Curcumin and nano-curcumin supplementation	↓
Boshagh et al. (2023) [208]	Patients with ischemic stroke in the rehabilitation stage	Curcumin–piperine supplementation	↓
Shafabakhsh et al. (2020) [209]	Patients with type 2 diabetes mellitus	Curcumin intake	↓
Santana-Garrido et al. (2020) [212]	Hypertensive mice	Wild olive (Acebuche) oil-enriched diet	↓
Ivanov et al. (2018) [213]	Spontaneously hypertensive rats	*Olea europaea L*. leaf extract	↓
Coutinho et al. (2017) [214]	LDLr−/− mice	Virola oleifera (Schott) A.	↓
Mautone Gomes et al. (2023) [215]	Wistar rats during metabolic syndrome	Coconut oil long-term supplementation	=
Liu et al. (2017) [216]	Rats	Daily sesame oil supplementation	↓
Al-Okbi et al. (2018) [217]	Rats	Borage and fish oil	↓
Perrone et al. (2019) [218]	Human	Hydroxytyrosol and derivatives, contained in extra virgin olive oil, typically used in Mediterranean diet	↓
Ghorbel et al. (2015) [219]	Rats with cardiotoxicity and DNA damage	Extra virgin olive oil and its lipophilic fraction and hydrophilic fraction	↓

The table summarizes findings from multiple studies analyzing the effects of different diets and antioxidant supplementations on oxidative stress modulation. “↑” denotes an increase, “↓” denotes a decrease, “=“ denotes no change.

## Data Availability

No new data were created or analyzed in this study.

## Abbreviations

AMI	acute myocardial infarction
BS	Behçet’s syndrome
CV	cardiovascular
CVD	cardiovascular disease
DASH	dietary approaches to stop hypertension
FORD	free oxygen radicals defense
FORT	free oxygen radicals test
GPx	glutathione peroxidase
GSH	glutathione
H_2_O_2_	hydrogen peroxide
HFHC	high-fat high-cholesterol
hs-CRP	high-sensitivity C-reactive protein
ICAM-1	intercellular adhesion molecule 1
IL-6	interleukin-6
KD	ketogenic diet
MCP-1	Monocyte Chemoattractant Protein-1
MD	Mediterranean diet
MDA	malondialdehyde
MetS	metabolic syndrome
MI	myocardial infarction
NADPH	nicotinamide adenine dinucleotide phosphate
NAFLD	non-alcoholic fatty liver disease
NEAC	non-enzymatic antioxidant capacity
NETs	neutrophil extracellular traps
NF-κB	nuclear factor-kappa B
NO	nitric oxide
NQO1	NADPH quinone dehydrogenase 1
O_2_^−•^	superoxide anion
OH^∙^	hydroxyl radical
ONOO^−^	peroxynitrite anion
ONOO^−^	peroxynitrite anion
PGI2	prostacyclin
PTMs	post-translational modifications
PUFAs	polyunsaturated fatty acids
RBCs	red blood cells
RCS	reactive carbon species
RNS	nitrogen species
RO^•^	alkoxyl radical
ROO^•^	peroxyl radical
ROOH	organic hydroperoxide
ROS	reactive oxygen species
RSeS	and reactive selenium species
RSS	reactive sulfur species
SLE	systemic lupus erythematosus
SOD	superoxide dismutase
T2D	type 2 diabetes
TAC	total antioxidant capacity
TF	tissue factor
TFPI	tissue factor pathway inhibitor
TLR	toll-like receptor
TM	thrombomodulin
TNF-α	tumor necrosis factor-alpha
VCAM-1	vascular cell adhesion molecule 1
vWF	von Willebrand factor
β-HB	β-hydroxybutyrate
